# Immunogenic cell death in cancer: concept and therapeutic implications

**DOI:** 10.1186/s12967-023-04017-6

**Published:** 2023-03-02

**Authors:** Lorenzo Galluzzi, Oliver Kepp, Erik Hett, Guido Kroemer, Francesco M. Marincola

**Affiliations:** 1 Department of Radiation Oncology, Weill Cornell Medical College, New York, NY USA; 2 Sandra and Edward Meyer Cancer Center, New York, NY USA; 3 Caryl and Israel Englander Institute for Precision Medicine, New York, NY USA; 4 Metabolomics and Cell Biology Platforms, Institut Gustave Roussy, Villejuif, France; 5 Sonata Therapeutics, Boston, MA USA; 6 Centre de Recherche des Cordeliers, Equipe labellisée par la Ligue contre le cancer, Université de Paris, Institut Universitaire de France, Sorbonne Université, Inserm U1138, Paris, France; 7 Institut du Cancer Paris CARPEM, Department of Biology, Hôpital Européen Georges Pompidou, AP-HP, Paris, France; 8 Kite Pharma Inc, Santa Monica, CA USA

**Keywords:** Antigenicity, CAR T cells, DAMPs, Immune checkpoint inhibitors, Pattern recognition receptors, Tumor microenvironment

## Abstract

Mammalian cells responding to specific perturbations of homeostasis can undergo a regulated variant of cell death that elicits adaptive immune responses. As immunogenic cell death (ICD) can only occur in a precise cellular and organismal context, it should be conceptually differentiated from instances of immunostimulation or inflammatory responses that do not mechanistically depend on cellular demise. Here, we critically discuss key conceptual and mechanistic aspects of ICD and its implications for cancer (immuno)therapy.

## Introduction

All mammalian cells (including normal and neoplastic cells) respond to relatively mild perturbations of homeostasis by activating signal transduction cascades aimed at repairing macromolecular and/or organellar damage and restoring normal cellular functions [1–4]. When successful, such stress responses fully re-establish cellular homeostasis, hence preserving organismal fitness [5, 6]. Conversely, failed adaptation to stress generally elicits regulated cell death (RCD) as a means to preserve organismal homeostasis in the context of cellular loss [7–9].

Importantly, most (if not all) cellular responses to stress are hard-wired to immune signaling [10]. Thus, even when normal cellular functions are ultimately restored, stressed cells pre-alert the immune system of a potential danger by: (1) altering their surface properties, and (2) releasing cytokines, chemokines and so-called damage-associated molecular patterns (DAMPs) [11–13]. Generally, these signals support the establishment of an inflammatory response that recruits innate immune effector cells to sites of cellular stress, but *per se* fail to elicit antigen-specific adaptive immunity [10]. Such an immune engagement, however, serves as a platform for the *potential* initiation of adaptive immune responses if stressed cells fail to recover homeostasis and ultimately undergo RCD [2, 14]. Whether RCD ultimately promotes or inhibits antigen-specific immune responses depends on several critical determinants [15, 16].

Here, we discuss key determinants of immunogenic cell death (ICD) and provide a brief overview of accumulating data on the prominent implications of ICD for cancer (immuno)therapy.

## Core ICD determinants

**Fig. 1:**
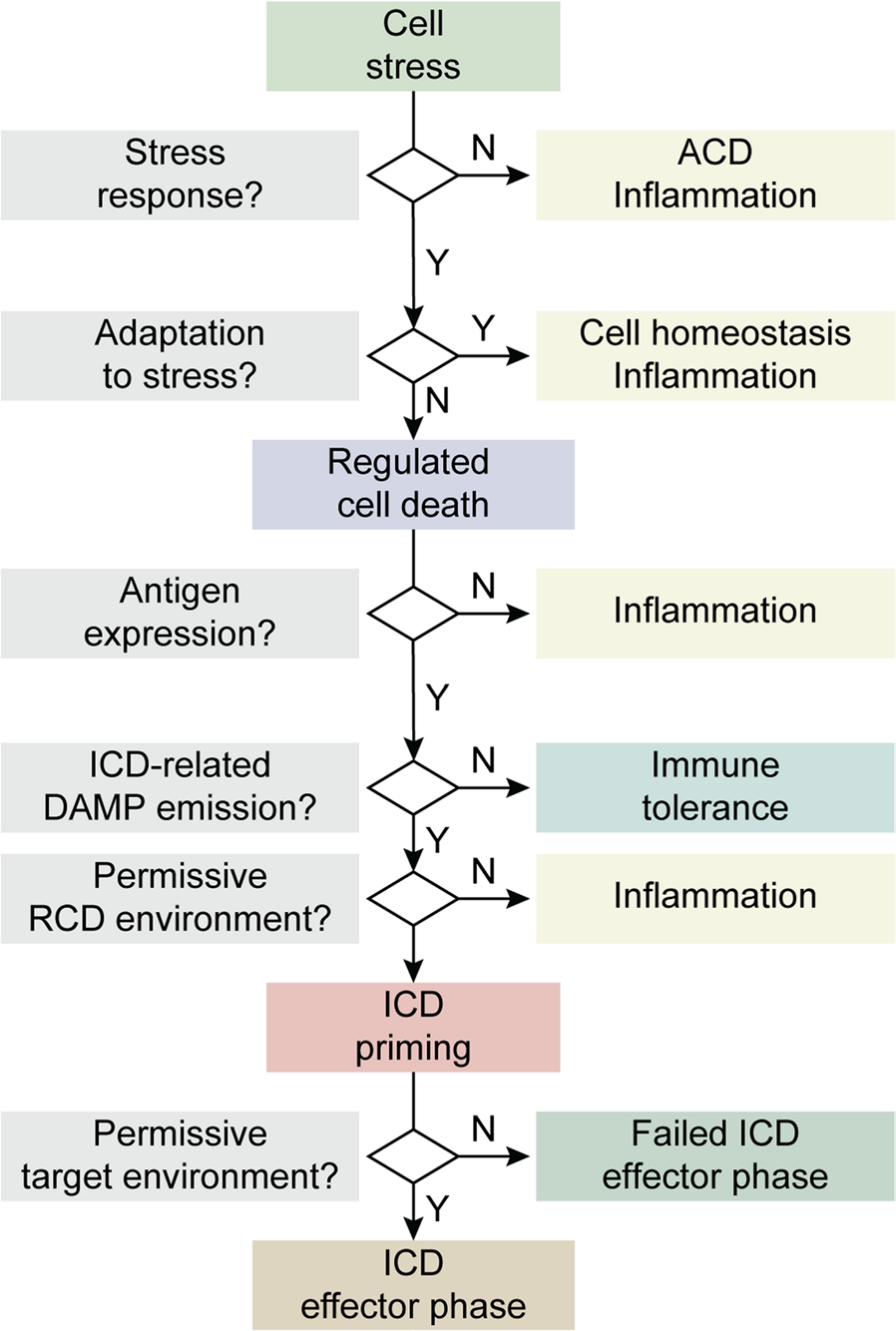
Core requirements for the initiation of adaptive immune responses by dying cells. For cell death to drive *bona fide* adaptive immune responses: (1) cell death must occur in the context of adaptive stress responses; (2) cell death must ultimately occur, as opposed to successful adaptation to stress; (3) dying cells must present antigens that are not covered by thymic tolerance; (4) regulated cell death (RCD) must be accompanied by the emission of endogenous molecules that operate as immunological adjuvants; and (5) microenvironmental conditions must be permissive for antigen-presenting cell (APC) recruitment, maturation and migration to lymph nodes (or other sites of antigen presentation), as well as for cytotoxic T lymphocyte (CTL) infiltration and activation. Depending on which of these conditions is lacking, cell death can drive innate immune signaling coupled with local inflammation, actively promote immunological tolerance and/or result in antigen-specific CTL priming and expansion but no effector immune response. ACD, accidental cell death; DAMP, damage-associated molecular pattern; ICD, immunogenic cell death

Five core features are required for RCD to elicit antigen-specific immune responses (over mere innate immune signaling coupled to inflammation) of relevance for cancer (immuno)therapy (Fig. 1). As discussed here below, the absence of any of these determinants converts ICD into immunologically silent or even tolerogenic variants of RCD.

### Stress

Cell death is not immunogenic when it occurs as an accidental, unregulated process that does not involve adaptation to stress, as in the presence of very harsh physicochemical or mechanic conditions (which can be modeled in experimental settings, but are quite rare in human pathophysiology) [17]. In line with this notion, cancer cells succumbing to a variety of therapeutic agents including selected chemotherapies [18], targeted anticancer agents [19] and radiation therapy (RT) [20] can be successfully used to elicit prophylactic anticancer immunity upon inoculation in immunocompetent, syngeneic hosts. However, the same does not hold true when the same cells are killed instantaneously by freeze-thawing cycles [21, 22]. Interestingly, although accidental cell death (ACD) occurring in the absence of stress responses results in a necrotic morphology that has been consistently associated with inflammation in patients affected by a variety of conditions, rapid ACD may turn out to be considerably less inflammatory than stress-driven regulated instances of necrosis such as necroptosis or pyroptosis [23]. Indeed, many of the immunostimulatory signals underlying inflammatory responses to necrotic cells are actively synthesized during stress responses (e.g., cytokines, chemokines) or released along with (failing) adaptation to stress (e.g., DAMPs) [13].

### Death

As mentioned above, cell death must occur for perturbations of cellular homeostasis to ultimately results in adaptive immune responses [15]. Thus, while successful adaptation to stress may still ignite local inflammatory responses, cancer cells must die for their corpses to be efficiently taken up by antigen-presenting cells (APCs), especially dendritic cells (DCs), and processed for antigen presentation [24, 25]. At least in part, this reflects the notion that immature DCs are highly proficient at (macro)pinocytosis, which involves material of subcellular size, but much less so at engulfing entire cells [26]. While macrophages are surely more efficient than DCs at the latter, (1) living cancer cells tend to express high levels of anti-phagocytic molecules such as CD47 [27, 28], but much less so pro-phagocytic molecules such as calreticulin (CALR) [29], on their surface; (2) macrophages generally take up cells and their corpses in an immunologically silent manner [30, 31]; (3) macrophages have limited migratory capacity and hence do not reach lymph nodes and generally are excluded by intratumoral tertiary lymphoid structures (another site of efficient antigen presentation to T cells) [32].

### Antigenicity

Cancer (and normal) cells undergoing stress-driven RCD must be sufficiently antigenic to elicit adaptive immune responses [33]. This means that dying cells must express antigens whose cognate T-cell receptor (TCR) has not be purged by the circulating T-cell repertoire during thymic selection [34]. The source of such antigenicity can vary quite considerably as it encompasses (1) pathogen-encoded antigens [15, 35], (2) mutational neoantigens [36], and (3) a large and hitherto poorly recognized panel of non-mutational neoantigens as generated, for instance, by epigenetic alterations resulting in transcriptional shifts [37], alternative splicing events [38], enzymatic and non-enzymatic protein modifications [39], and/or translation of cryptic sequences [40]. Thus, irrespective of antigen source, RCD can be immunogenic in one host but not necessarily in another, simply reflecting interindividual differences in the circulating T-cell repertoire [41]. In the absence of antigenicity, stress-driven RCD causes robust inflammatory reactions that are relevant for a variety of non-malignant disorders [42], but it fails to engage adaptive immune modules.

### Adjuvanticity

Similar to prophylactic vaccines against pathogens, ICD requires robust adjuvants to initiate adaptive immune responses [43, 44]. Such adjuvants, which are commonly referred to as DAMPs, are fully endogenous to dying cells and are generally released or exposed on the plasma membrane as a consequence of pre-mortem cellular stress [45]. DAMPs can be broadly classified into three main families: pro-phagocytic signals, immunostimulatory molecules and cytokines/chemokines [46]. The prototypic ICD-associated “eat-me” signal is CALR, an endoplasmic reticulum (ER) chaperone that is exposed on the outer leaflet of the plasma membrane downstream of the integrated stress response (ISR) and consequent phosphorylation of eukaryotic translation initiation factor 2 subunit alpha (EIF2S1, best known as eIF2α) [47, 48]. Common immunostimulatory DAMPs mechanistically linked to ICD encompass ATP, which is actively secreted by an autophagy-dependent mechanism [49], as well as high mobility group box 1 (HMGB1) and annexin A1, both of which appear to be passively released upon nuclear and plasma membrane permeabilization [50–52]. Finally, type I interferon (IFN) and C-X-C motif chemokine ligand 10 (CXCL10) have been involved in multiple instances of ICD [53, 54]. Of note, multiple DAMPs operate by binding to pattern recognition receptors (PRRs) expressed on immune cells that originally evolved as part of the host defense from pathogens, such as Toll-like receptor 4 (TLR4), which binds HMGB1 [46], and formyl peptide receptor 1 (FPR1), which binds ANXA1 [52]. Thus, not only defects in DAMP emission, but also lack or dysfunction of cognate PRRs can abolish the immunogenicity of RCD. Importantly, in the absence of adjuvanticity, the stress-driven demise of cells with sufficient antigenicity actively drives DC-dependent immune tolerance [10].

### Microenvironment

There is an important microenvironmental component in the elicitation of adaptive immunity by cancer cells undergoing RCD [55]. On the one hand, the microenvironment of dying cells must be permissive for infiltration by APC precursors, their maturation/activation and either their egress to draining lymph nodes or their incorporation into tertiary lymphoid structures for local antigen presentation to T cells [56]. Thus, while in prophylactic experimental settings (that involve the subcutaneous administration of cancer cells exposed to ICD-inducing agents in vitro) the dermis offers a privileged, fully immunocompetent microenvironment for the elicitation of adaptive immunity (provided that all other core ICD determinants are present) [43, 57], the same may not always hold true when RCD occurs within the TME, which is generally dominated by immunosuppressive mechanisms that may interfere with APC functions [58, 59]. On the other hand, antigen specific T cells as efficiently primed by ICD-elicited APCs must have access to their targets and encounter favorable conditions for mediating effector functions [58, 60]. This implies that even in the context of robust T cell priming and clonal expansion, malignant lesions may be protected from immunological eradication as a consequence of stromal exclusion and/or local immunosuppression, for instance upon direct T cell inhibition via CD274 (best known as PD-L1).

Taken together, these observations delineate the key molecular and cellular components of adaptive immune responses elicited by ICD, as opposed to innate immune signaling and inflammation as driven by non-immunogenic RCD variants. Supporting the central relevance of each of these mechanisms, both pathogens and malignant cells have evolved a variety of strategies to either subvert immunogenic stress signaling, RCD, antigenicity and/or adjuvanticity, or condition the microenvironment to suppress ICD initiation or execution [15, 61]. Discussing these strategies in detail, however, goes largely beyond the scope of the present Commentary.

## ICD and cancer (immuno)therapy

Accumulating preclinical and clinical data suggest that the induction of ICD is particularly relevant for the efficacy of cancer (immuno)therapy [62].

### Preclinical evidence

In a variety of rodent tumor models, ICD signaling has been mechanistically linked to superior responses to clinically relevant therapies, including (but not limited to) chemotherapy based on anthracycline and (some) platinum derivatives [63, 64], targeted anticancer agents specific for epidermal growth factor receptor (EGFR) [19], multitarget tyrosine kinase inhibitors [65], radiation therapy [66, 67], and photodynamic therapy [68]. Specifically, in numerous prophylactic or therapeutic experimental settings involving the aforementioned clinically relevant agents, pharmacological or genetic strategies interrupting stress signaling in cancer cells, DAMP emission therefrom, or DAMP detection by immune cells compromised the emergence of protective anticancer immunity or disease control, respectively [45]. Similar defects in prophylactic or therapeutic disease control have been documented upon the depletion or inhibition of numerous immune effector cells involved in the elicitation of anticancer immunity downstream of ICD, such as DCs [47], interleukin 17 A (IL17A)-producing γδ T cells [69], as well as CD4^+^ and CD8^+^ T cells [70]. Importantly though, documenting a drop in treatment efficacy in tumor-bearing mice subjected to pharmacological or genetic strategies that block specific immune functions as compared to their fully immunocompetent counterparts does not necessarily identify *bona fide* ICD induction [15]. Along similar lines, while a wide panel of *bona fide* ICD inducers have been shown to synergize (or at least positively interact) with immune checkpoint inhibitors (ICIs) in otherwise ICI-resistant mouse tumor models [71], the formal implication of ICD in these findings remains to be formally elucidated. Indeed, multiple anticancer agents exert therapeutically relevant immunostimulatory effects that are RCD-independent and rather reflect the direct interactions between such agents and vascular, stromal, immunological or microbial components of the local or systemic TME [72]. This latter consideration largely justifies prophylactic vaccination assays as a simple, widely applicable experimental approach to discriminate between *bona fide* ICD and the RCD-independent derepression of pre-existing (ICI-actionable) adaptive immune responses [15].

### Clinical evidence

At least three lines of correlative clinical evidence are available in support of the key relevance of ICD for cancer (immuno)therapy. First, in numerous cohorts of patients with cancer, defects in immunogenic stress signaling, RCD, DAMP emission or DAMP sensing have been shown to have a detrimental impact not only on prognosis in largely unselected patient populations [52], but also on response to ICD-inducing therapeutic agents [51]. Such defects encompass molecular or transcriptional signatures of suboptimal cellular responses to stress (e.g., poor eIF2α phosphorylation) [73], reduced expression levels of specific DAMPs or receptors thereof (e.g., low CALR expression) [74], as well as single-nucleotide polymorphisms associated with limited PRR signaling (in *TLR4* or *FPR1*, for instance) [51, 52].

Second, a considerable fraction of the therapeutic armamentarium currently available for clinical cancer management has been shown to elicit ICD (or other forms of immunostimulation) [75]. Importantly, these approaches have often been developed into clinically efficient therapies in an empirical and immune agnostic manner (i.e., harnessing human cancer xenografts in immunodeficient mice at preclinical stages and developing therapeutic schedules in patients via the maximum tolerated dose paradigm) [76]. Thus, if (ICD-driven) anticancer immunity had relevance for therapeutic outcome, one would expect immunostimulatory agents (including ICD inducers) to be enriched as compared to immunosuppressive (or immunologically neutral) therapies, which currently is the case [10]. Moreover, drug discovery programs have been designed to actively search for ICD inducer and two of such drugs, i.e., lurbinectedin and belantamab mafodotin, have received regulatory approval for use in cancer patients [77, 78].

Third, in line with preclinical findings, a growing number of ICD inducers positively interact with ICIs or other immunotherapeutic approaches in patients with cancer [79, 80]. Notable examples of such successful combinations include (1) nab-paclitaxel plus atezolizumab (an ICI specific for PD-L1), which is currently employed in the management of triple negative breast cancer (TNBC) [81], carboplatin/etoposide plus atezolizumab, which is approved for patients with extensive-stage small cell lung cancer (SCLC) [82], as well nab-paclitaxel/carboplatin plus the programmed cell death 1 (PDCD1, best known as PD-1) blocker pembrolizumab [83].

Altogether, these preclinical and clinical findings suggest that ICD induction plays a major role in the successful control of multiple neoplasms by (immuno)therapy.

## Conclusions and future perspectives

In summary, ICD-driven adaptive immunity is mechanistically and conceptually different from both inflammatory reactions driven by non-immunogenic variants of RCD and adaptive immune responses that do not rely on cell stress and death. Importantly, several RCD routines have been characterized in molecular terms and classified based on the mechanistic involvement of specific signal transduction cascades (Table 1) [9]. For instance, apoptosis is currently defined as an RCD variant that is precipitated by the activation of cysteine proteases of the caspase family, while necroptosis involves the activating phosphorylation of receptor interacting serine/threonine kinase 3 (RIPK3) and consequent phosphorylation-dependent oligomerization of the pore-forming protein mixed lineage kinase domain like pseudokinase (MLKL) [84]. That said, once adaptation to stress fails, cells appear to die irrespective of active signaling, largely because of bioenergetic failure and/or irreparable damage to macromolecular structures that underlie cellular homeostasis itself, including (but not limited to) organelles and membranes [9]. The signal transduction cascades elicited during cell death rather seem to determine the kinetic and immunological manifestations of the process, rather than its occurrence *sensu stictu* [85]. In line with this notion, both pharmacological and genetic interventions targeting so-called “executioners” of cell death invariably delay the cellular demise, but do not prevent it, at least in mammalian systems [9].

**Table 1:** Key aspects of regulated cell death variants

Mode	Morphology	Prototypic inducers	Core mediators	Note(s)
ADCD	Autophagic	n.d.	Autophagy	May involve components of the autophagy machinery rather than *bona fide* lysosomal degradation
Entotic cell death	Entotic	Cell detachment	Actomyosin Lysosomes	Independent of phagocytic activity in engulfing cells
Extrinsic apoptosis	Apoptotic	Death receptor signaling	CASP8 CASP3	Negatively regulated by CFLAR and XIAP, sometimes involving MOMP
Ferroptosis	Necrotic	Erastin	ACSL4 LPCAT3	Iron-dependent, negatively regulated by GPX4 and FSP1
Intrinsic apoptosis	Apoptotic	DNA damage ER stress	BAK1 BAX CASP3	Demarcated by MOMP and negatively regulated by antiapoptotic BCL2 proteins
LDCD	Necrotic	Lysosomotropic agents	Cathepsins	Demarcated by primary LMP
MPT-driven necrosis	Necrotic	Ca2 + overload Oxidative stress	PPIF	Demarcated by MIMP and involving a poorly characterized multiprotein pore (PTPC)
Necroptosis	Necrotic	Death receptor signaling plus caspase inhibition Viral infection	RIPK1 RIPK3 MLKL	Negatively regulated by CASP8 and ADAR1
NETotic cell death	Necrotic	Pathogen infection	NADPH oxidases	Associated with NET extrusion
Parthanatos	Necrotic	PARP1 hyperactivation	AIFM1	Reflecting lethal NAD^+^ and ATP depletion

*ADCD* autophagy-dependent cell death,* LDCD* lysosome-dependent cell death,* LMP* lysosomal membrane permeabilization,* MIMP* mitochondrial inner membrane permeabilization,* MOMP* mitochondrial outer membrane permeabilization,* MPT* mitochondrial permeability transition,* n.d.* not determined,* NET* neutrophil extracellular trap,* PTPC* permeability transition pore complex

*Limited to main regulated cell death (RCD) modalities, adapted from Ref. [9]

Most importantly, the biochemical cascades underlying RCD in its multiple variants are not necessarily linked to its immunogenicity [85]. As a standalone example, apoptotic cell death as precipitated by caspases is normally an immunologically silent event, largely reflecting the ability of caspase 3 (CASP3) to initiate signaling pathways that promote macrophage-mediated efferocytosis in the absence of active immunostimulatory signaling and the overall implication of apoptosis in development and adult tissue homeostasis [86]. However, multiple caspase-dependent instances of RCD that classify as ICD by all definitions have been reported [22, 87]. Thus, the immunogenicity of a specific RCD instance cannot be determined with certainty based on the molecular pathways that precipitate RCD only, as abundantly discussed herein. Indeed, RCD-independent, host-related factors including antigenicity and microenvironmental parameters stand out as critical determinants of RCD immunogenicity [10].

Despite this and other conceptual (and experimental) caveats, ICD stands out as a major, therapeutically actionable process for cancer immuno(therapy). Future efforts will have to focus on identifying novel, clinically useful ICD inducers (irrespective of the RCD mode they impinge on) as well as biomarkers predicting the likelihood of specific neoplastic lesions to elicit adaptive immune responses downstream of ICD in response to treatment. Alongside, it will be important to devise clinically viable strategies to increase the immunogenicity of otherwise immunologically silent RCD variants, and to investigate novel combinatorial regimens combining ICD inducers and immunotherapy in the clinic, with the ultimate goal to facilitate efficient anticancer immunosurveillance. We surmise that ICD induction will occupy an ever more central stage in modern cancer management.

## Data Availability

Not applicable.
